# Robust hierarchical 3D carbon foam electrode for efficient water electrolysis

**DOI:** 10.1038/s41598-017-05215-1

**Published:** 2017-07-21

**Authors:** Tung Ngoc Pham, Tiva Sharifi, Robin Sandström, William Siljebo, Andrey Shchukarev, Krisztian Kordas, Thomas Wågberg, Jyri-Pekka Mikkola

**Affiliations:** 1 Technical Chemistry, Department of Chemistry, Chemical-Biological Centre, Umeå University, SE-90187 Umeå, Sweden; 2 Department of Chemistry, The University of Danang, University of Science and Technology, 54 Nguyen Luong Bang, Lien Chieu, Danang Vietnam; 3 Department of Physics, Umeå University, SE-90187 Umeå, Sweden; 4 Microelectronics Research Unit, University of Oulu, P.O. Box 4500, FI-90014 University of Oulu, Oulu, Finland; 5 Industrial Chemistry & Reaction Engineering, Department of Chemical Engineering, Process Chemistry Centre, Åbo Akademi University, FI-20500 Åbo-Turku, Finland

## Abstract

Herein we report a 3D heterostructure comprising a hierarchical macroporous carbon foam that incorporates mesoporous carbon nanotubes decorated with cobalt oxide nanoparticles as an unique and highly efficient electrode material for the oxygen evolution reaction (OER) in electrocatalytic water splitting. The best performing electrode material showed high stability after 10 h, at constant potential of 1.7 V vs. RHE (reversible hydrogen electrode) in a 0.1 M KOH solution and high electrocatalytic activity in OER with low overpotential (0.38 V vs RHE at 10 mA cm^−2^). The excellent electrocatalytic performance of the electrode is rationalized by the overall 3D macroporous structure and with the firmly integrated CNTs directly grown on the foam, resulting in a large specific surface area, good electrical conductivity, as well as an efficient electrolyte transport into the whole electrode matrix concurrent with an ability to quickly dispose oxygen bubbles into the electrolyte. The eminent properties of the three-dimensional structured carbon matrix, which can be synthesized through a simple, scalable and cost effective pyrolysis process show that it has potential to be implemented in large-scale water electrolysis systems.

## Introduction

The imminent threat of global warming and climate change brought by carbon dioxide associated with the extensive use of fossil fuels has turned academic and industrial attention towards hydrogen, known as a clean fuel and could function as an excellent alternative to traditional fossil fuels^1–4^. Water electrolysis is one of the most important non-polluting methods to obtain hydrogen from water, in particular when coupled to a renewable energy source such as solar energy^5–8^. The water electrolysis technology is based on the generation of hydrogen at cathode and oxygen at anode by passing an electric current through water. One of the biggest problems in the electrocatalytic water splitting process is the sluggish kinetics frequently observed for the oxygen evolution reaction (OER) on the anode^7, 9^. Today, ongoing research efforts focus on the development of effective catalysts in order to speed up the reaction rate, lower the overpotential and to exhibit good stability. Noble metal oxide based catalysts such as IrO_2_ and RuO_2_ have a documented high electrocatalytic activity in OER^7, 10^ however the high cost and scarcity of these noble metals limit their application, especially in large scale. Consequently, transition metal oxide catalysts based on non-precious and more abundant metals such as iron, nickel, cobalt and manganese have been proposed as viable alternatives to noble metals^11–14^. Among them, cobalt oxide (CoO_x_) catalyst have been widely studied as an alternative for noble metals based catalysts due to its low cost, low environmental foot print and good catalytic activity in OER^11, 15, 16^.

Upon electrode fabrication, powder form CoO_x_ is typically attached onto conductive substrates using polymeric binders such as Nafion^®^. However, the employment of any binder can deteriorate the overall performance of the catalyst by (1) reducing the contact area between the electrolyte and active sites; (2) limiting carrier transport within the electrode; (3) hampering electrode stability, thus resulting in compromised electrocatalytic performance of the electrode^12, 17^. To further improve the electrode characteristics, especially considering the poor conductivity of most metal oxides, an intimate integration of metal oxide nanoparticles with different substrates such as mesoporous silica, nickel foam and carbon materials have been developed^11, 15–17^, with a focus on achieving a good electrical conductivity and high surface area. More recently, 3D electrodes fabricated from materials such as nickel foam, graphene, carbon cloth and carbon paper have been developed to retain all above characteristics as well as to allow for swift and unhindered penetration of electrolytes into the whole electrode matrix^11, 12, 16, 18^. Further development of such electrode materials that are flexible, low-weight and that can be produced from abundant materials by methods that enable upscaling is thus highly motivating.

Here we introduce a new hierarchically 3D structured, low-weight carbon foam with high surface area and high compressibility, synthesized directly from a commercially available, low-cost melamine foam (Supplementary Fig. S1a)^19^. Additionally, due to the simple and cost effective synthesis methods (pyrolysis and activation), the melamine based carbon foam has great potential for large-scale applications. Comparing to other carbon foam materials *e.g*. carbon paper and carbon aerogel, these above-mentioned characteristics indicate that the melamine based carbon foam has at least equal or even better potential to be used as electrode material for OER. The most conductive carbon foam introduced in our previous study^19^ (denoted as A800) also possesses the highest surface area and was used as a reference material in this study. Herein we demonstrate that the new carbon foam (denoted as P900) exhibits excellent properties as a hybrid electrode for oxygen evolution reactions (OER). The material was obtained after a heat treatment process to increase its electrical conductivity and carbon nanotubes (CNTs) were grown in the pores and on the surface of P900 to further increase its surface area. Finally, after subsequent decoration of the CNTs/P900 support by CoO_x_ nanoparticles a material with excellent properties was obtained. We further show that despite heat treatment and integration of CNTs into the carbon foam, the overall scaffold-like structure with its flexible characteristics is fully preserved. The (CoO_x_@CNTs/P900) electrode could be implemented directly as for OER and displays a low overpotential of 0.38 V vs RHE at 10 mA cm^−2^ and a good stability (~10 h at constant potential of 1.7 V vs RHE) in a 0.1 M KOH solution.

## Experimental

### Materials

Melamine foam (Basotect^®^ G) was purchased from BASF. Cobalt (II) acetate tetrahydrate (Co(C_2_H_3_O_2_)_2_. 4H_2_O) and thiophene (C_4_H_4_S, 99%) were purchased from Sigma Aldrich. Silver paint was purchased from PELCO^®^. All chemicals were used as received.

### Synthesis of carbon foam

The activated carbon foam sample (denoted as A800, where A stands for activated and the number denotes the treatment temperature) was synthesized following a procedure reported in ref. 18. Briefly, melamine-based polymer foam (BASF, Basotect® G, used as received) was pyrolyzed at 800 °C (1 hour with the ramping rate of 1 °C/min, under N_2_ flow (50 ml/min)) in a quartz reactor. Immediately after the pyrolysis, an activation gas mixture (2% CO_2_ in N_2_, 50 ml/min) was introduced into the system for 2 hours at the same temperature (at 800 °C). On the other hand, P900 sample (where P stands for pyrolyzed and the number denotes the treatment temperature) was produced by the pyrolysis of the polymer foam at 900 °C (6 hours, the ramping rate of 5 °C/min) in a quartz reactor under N_2_ flow (50 ml/min). After completed heat treatment and activation process, the system was allowed to cool to room temperature under inert (nitrogen) atmosphere.

### Synthesis of CNTs on carbon foam

CNTs were grown on the carbon foam substrate by means of catalytic chemical vapor deposition (CCVD). In this study, only cobalt decorated P900 sample was used as a substrate to grow CNTs. The sample was placed on a quartz boat which then was inserted into a horizontal quartz tube. The system was purged with the Varigon gas (5% hydrogen in argon gas, 180 mL/min) for 20 minutes and then heated to 670 °C with 20 min heating time. When the desired temperature was reached, acetylene was introduced into the system (~3.8 ml/min) for 30 minutes (while the Varigon gas flow was also maintained). The system was then allowed to cool down to room temperature under argon atmosphere (180 mL/min). The final product (CNTs/P900) was stored in Falcon tubes for further use.

### Synthesis of catalyst material

In a typical process, 20 mg of cobalt (II) acetate tetrahydrate was dissolved in 5 ml of dimethyl formamide (DMF) and sonicated for 3 minutes. The carbon foam sample (20 mg) which can be either A800 or P900 or CNTs/P900, was submerged into the mixture together with 65 μL of thiophene. The mixture was then sonicated for 20 minutes, followed by a drying at 125 °C under nitrogen flow. Finally, the obtained sample was annealed at 400 °C for 2 h in nitrogen atmosphere to facilitate the formation of CoO_x_. Depending on the initial carbon foam (A800, P900 and CNTs/P900), the final products were denoted as CoO_x_@A800, CoO_x_@P900 and CoO_x_@CNTs/P900, respectively. It is important to note that, a similar procedure (but without annealing step) was used to decorate cobalt on P900 which was later used to synthesis CNTs/P900.

### Characterization

Scanning electron microscopy (SEM) was carried out using a Zeiss Merlin FEG-SEM instrument. High-resolution transmission electron microscopy (HRTEM) image was obtained using a JEOL 2100 F instrument operating at 200 keV. Thermogravimetric analysis (TGA) was conducted on a Mettler Toledo equipment (TGA/DSC 1LF) operated at a heating rate of 10 °C/min up to 950 °C in air. The surface chemistry of the samples was examined by the means of X-ray photoelectron spectroscopy (XPS). The photoelectron spectra were collected with a Kratos Axis Ultra DLD electron spectrometer using monochromated Al K_α_ source operated at 120 W. An analyzer pass energy of 160 eV for acquiring wide spectra and a pass energy of 20 eV for individual photoelectron lines were used. The surface potential was stabilized by the spectrometer charge neutralization system. Processing of the spectra was accomplished with the Kratos software. Raman spectroscopy was performed on a Renishaw InVia Raman spectrometer using a laser excitation wavelength of 633 nm. The CoO_x_ particle size distribution was determined by counting around 70 particles per sample based on SEM images. The content of cobalt element in the electrolyte was detected by using an Inductively Coupled Plasma Optical Emission Spectrometer (ICP/OES) Optima 2000 DV (Perkin Elmer Instruments).

The samples used in the current-voltage studies measurements were cut to a size of ~12 × 5 × 2 mm^3^ with a surgical blade. The samples were placed over copper electrodes made from a circuit board (electrode size of 24 × 3 mm^2^ and spacing of ~0.3 mm between adjacent electrodes). To ensure an intimate contact with the electrodes, the sponges were slightly pressed against the substrate by placing a weight (1.5 g) on the top. The copper electrodes were connected to a potentiostat (Metrohm Autolab, PGSTAT302N). Current-voltage sweeps were performed from −1.0 V to 1.0 V at scan rate of 0.1 V/s in air at room temperature. The resistance was estimated based on the I-V curve with 95% confidence interval.

To prepare electrodes, different electrode materials (A800, CoO_x_@A800, P900, CoO_x_@P900, CNTs/P900 and CoO_x_@CNTs/P900) were cut to an appropriate form from the bulks of the respective materials by a surgical blade. The thickness of samples was controlled at around 1.7 mm. A copper wire was attached to an electrode material using silver paste (Supplementary Fig. S1b) and allowed to dry overnight. A platinum coil and Ag/AgCl (1 M KCl) were used as the counter and reference electrodes, respectively, in a three-electrode electrochemical cell (Supplementary Fig. S1c). Linear sweep voltammery (LSV) was performed at a scan rate of 2 mV/s in a 0.1 M KOH solution. Note that 0.1 M KOH solution was used as electrolyte in all electrocatalytic activity tests including stability test in this study. For the stability test, chronoamperometry data were recorded at a constant potential of 1.7 V vs RHE. After 5 hours, the old electrolyte inside the electrochemical cell was replaced with a fresh electrolyte. Another type of stability test was also performed in this study where the sample was tested by 100 consecutive LSV scans (scan rate 5 mV/s). A potentiostat (Metrohm Autolab, PGSTAT302N) connected with FRA32M module (for impedance spectroscopy) was used for all of the electrochemical tests.

## Results and Discussions

**Figure 1:**
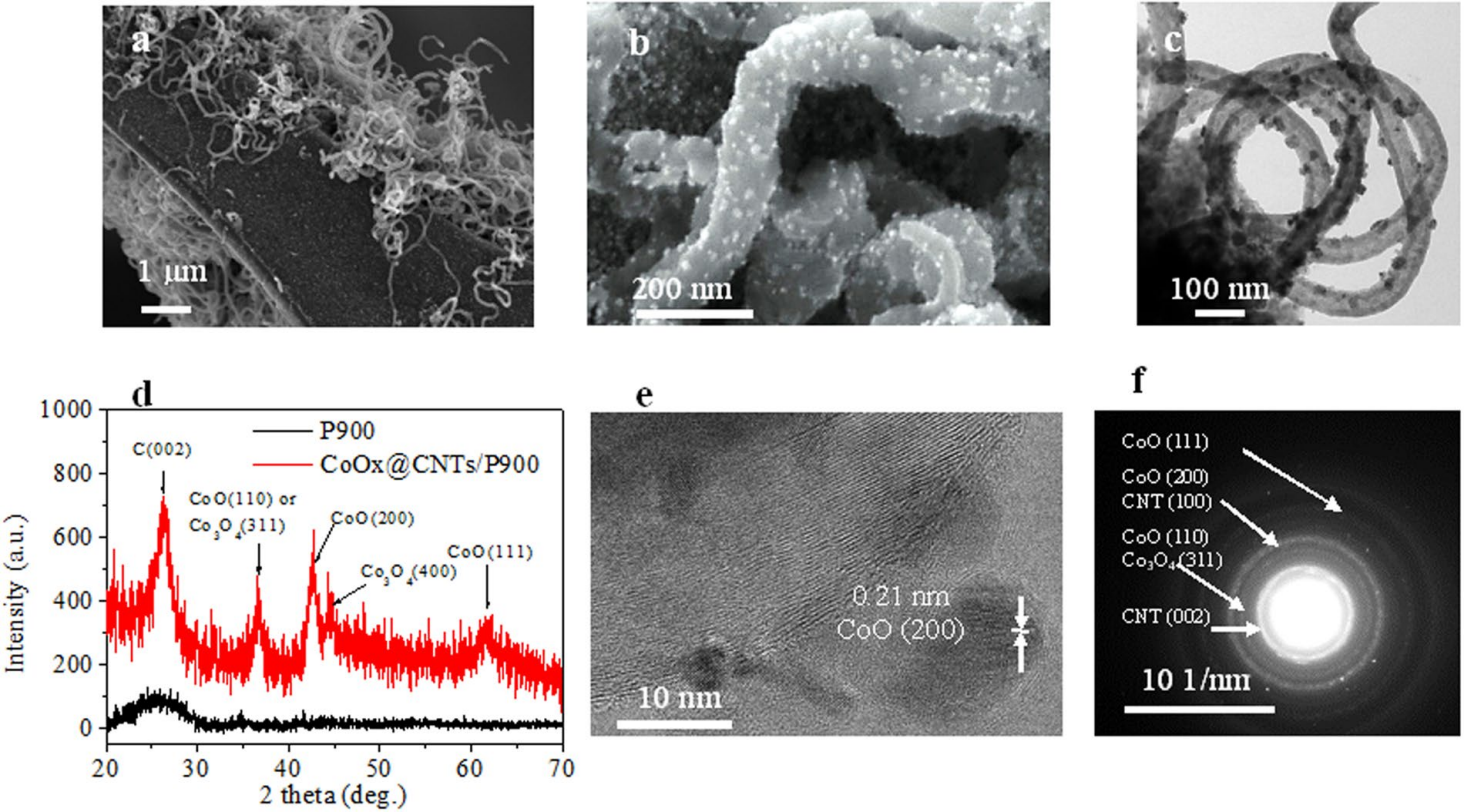
Structure and composition of the hierarchical carbon foam-nanotube structure decorated with CoO_x_ nanoparticles. (**a**) Low and (**b**) high magnification scanning electron micrographs, (**c**) TEM image and (**d**) XRD patterns of P900 and CoO_x_@CNTs/P900 sample, (**e**) HRTEM image and (**f**) Electron diffraction pattern of ‘fresh’ CoO_x_@CNTs/P900 electrode.

The results in Table 1 indicate that there is a relation between the electrical conductivity of the carbon foam samples and the carbon content on the surface which is in agreement with the study of Ramos *et al*. about the electrical properties of activated carbon cloths^20^. Our previous study^19^ also showed that the carbon content at the surface of carbon foam as well as their electrical conductivity are directly proportional with the pyrolysis temperature. For example, A800 which was pyrolyzed and activated at 800 °C was found as the most conductive carbon foam compared with other samples which were treated at lower temperature. Due to the electrical conductivity and the high surface area brought by the activation process, A800 was selected as a reference material in this study. On the other hand, as can be seen in the XRD pattern of P900 sample (Fig. 1d), a broad peak appears at 2θ ~26° which can be assigned to C (002) reflection. Thus, even though P900 is still lacking long-range crystal order as shown by the broadened C (002) reflection, it has higher graphitization degree than that of A800 showing no reflection at all for C (002) planes^19^. Higher treatment temperature for P900 sample can facilitate the formation of sp^2^ π bonded carbon volume fraction^21^ leading to dramatic increase of P900 conductivity compared with A800 sample. Moreover, the high amount of nitrogen found on the surface of A800 (more than 16 at. %, Table 1) can also hinder the charge transfer by the forming of insulating phase at high concentration of nitrogen^22, 23^. Additionally, due to the treatment at higher temperature and longer time than in the case of the A800 sample, a higher extent of shrinkage was observed for the P900 sample. In this sample, more than 90 wt. % and around 55% in terms of volume of the precursor polymer foam was lost after completion of the pyrolysis process (compared with around 80 wt.% and 40% in terms of volume for the A800 sample^19^). The increased shrinkage of the P900 sample resulted in a smaller sample void volume that could also contribute to a better electrical conductivity of P900 sample. As shown in Supplementary Fig. S2, the electrical conductivity of P900 is far better than that of A800. The estimated resistances of P900 and A800 are 518 ± 1 Ω and 64.7 ± 0.2 kΩ, respectively which is in agreement with the assumption above. Thus, based on these results we expected that the P900 would be the most promising material as the catalyst support for OER. Further evidence for this hypothesis will be presented in the following paragraph.

**Table 1:** Properties of different carbon foam samples.

	B.E.T surface area (m^2^/g)	Electrical resistance (kΩ)	Elemental analysis (at. %) by XPS		
			C	N	O
A800	>300	64.7 ± 0.2	73.1	16.4	8.7
P900	~4	0.518 ± 0.001	85.4	7.1	6.4
CNTs/P900	~120	0.412 ± 0.001	98.1	N/A*	1.4

*Below the detection limit.

**Figure 2:**
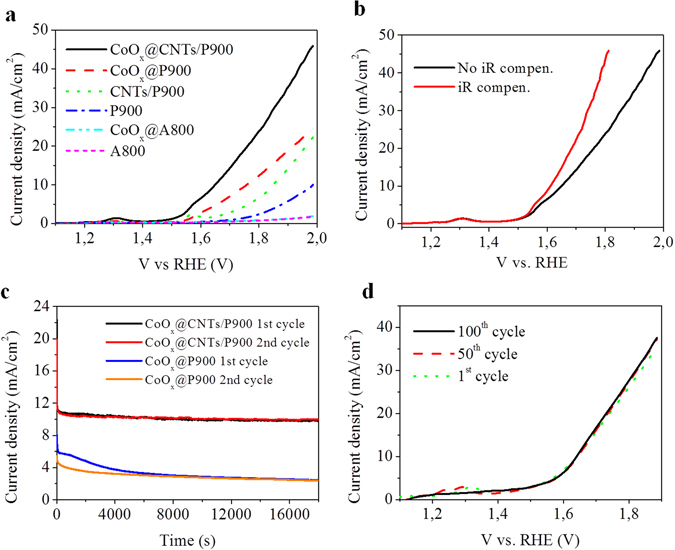
Electrochemical performance of the carbon foam-based electrodes. (**a**) polarization curves for OER on different carbon foam based electrodes at a scan rate of 2 mV/s, (**b**) Polarization curves of CoO_x_@CNTs/P900 under iR compensation at a scan rate of 2 mV/s (~19 ohms, identified by impedance spectroscopy, Complementary Fig. S9a). (**c**) Chronoamperometric reponses of CoO_x_@/P900 and CoO_x_@CNTs/P900 at a constant potential of 1.7 V vs. RHE and (**d**) 100 consecutive polarization scans obtained with CoO_x_@CNTs/P900 electrode (at a scan rate of 5 mV/s). All of the tests were performed in 0.1 M KOH solution.

The electrocatalytic activities of CoOx@A800 and CoOx@P900 together with “blank” reference (A800 and P900) samples were tested in alkaline electrolyte (0.1 M KOH). As shown in Fig. 2a, the bare P900 electrode has shown much better electrocatalytic activity compared with that of the bare A800 electrode which further confirmed for our initial conclusion in the previous paragraph. Moreover, at a lower nitrogen content (Table 1 and Supplementary Fig. S3a), some certain type of nitrogen such as pyridinic and quaternary nitrogen (Supplementary Fig. S3b) on the surface of P900 can also contribute to the OER performance of the sample^24, 25^. Cobalt oxide was successfully decorated on carbon foam samples, A800 and P900, as shown in Supplementary Figs S4 and S5a, respectively. At a similar catalyst loading (17 and 16 wt. % for CoO_x_@A800 and CoO_x_@P900, respectively), while finely dispersed cobalt oxide nanoparticles were formed on the CoO_x_@A800 surface, a semi-thin film of cobalt oxide was found on the surface of CoO_x_@P900. It is evident that the introduction of cobalt oxide on carbon foam gives rise to a better electrocatalytic activity with a lower overpotential and a higher anodic current. For example, the CoO_x_@P900 electrode demonstrated a higher anodic current (around 2.5 times higher than that of P900) and also a lower overpotential compared with the bare P900 electrode (0.53 V and 0.70 V, respectively). However, in the case of the A800 material, the introduction of the cobalt oxide could neither improve the overpotential nor the anodic current. Therefore, A800 is completely unsuitable for the application in question. The low electrocatalytic activity of A800 and CoO_x_@A800 also confirmed that the contribution of silver and copper (if any) to the performance of the electrodes is small. Generally, based on the electrocatalytic performance, we came up with a conclusion that the material P900 is the most suitable one to be used as an electrode in OER.

As discussed earlier, the high electrical conductivity of P900 renders it suitable as a catalyst support for OER. However, its low surface area (Table 1) limits the electrocatalytic performance. Thus, in order to increase the surface area, CNTs were directly grown on the P900 surface (Fig. 1 and Supplementary Fig. S6) using the CCVD method without destroying the carbon body of the foam. It is clear that the carbon foam was densely covered by CNTs with some parts being less covered (Fig. 1a) and some fraction could containing fiber-like material. The successful growth of well-entangled CNTs onto the carbon foam (P900) was manifested by an increase in the B.E.T. surface area from 4 m^2^/g for P900 to 122 m^2^/g for CNTs/P900. The electrical conductivity of the CNTs/P900 was slightly improved compared to P900 (Supplementary Fig. S2). The estimated resistances were 412 ± 1 and 518 ± 1 Ω for CNTs/P900 and P900, respectively. The measured impedance of both P900 and CNTs/P900 electrode materials in the frequency range of 0.01 to 105 Hz was shown as the Nyquist plots in the Supplementary Fig. S9b. As shown in the figure, the ohmic resistance of the electrolyte and the internal resistance of the electrode described as R_S_ were represented by the value at the intersection point on the real axis (Z’) at high frequency^26^. Thus based on the the R_S_ value of P900 and CNTs/P900 (which were estimated as 26.20 Ω and 19.07 Ω, respectively) we can conclude that the internal resistance of CNTs/P900 electrode is slightly beter than that of P900 electrode. This conclusion is also in agreement with the measured bulk resistance of these materials (Supplementary Fig. S2). On the other hand, in the case of CNTs/P900 electrode a stepper slope in the high frequency region, which also means a faster ion mobility than that of P900 electrode for the double layer formation^26^, was observed. The faster ion mobility could be facilitated by the higher electrical conductivity of the CNTs/P900 sample brought by the excellent electrical conductivity of CNTs. Figure 1b showed that after applying the capping agent (thiophene)^27^ a homogenous decoration of CoO_x_ nanoparticles in the range of 4–14 nm (Supplementary Fig. S7) was achieved (CoO_x_@CNTs/P900).

**Figure 3:**
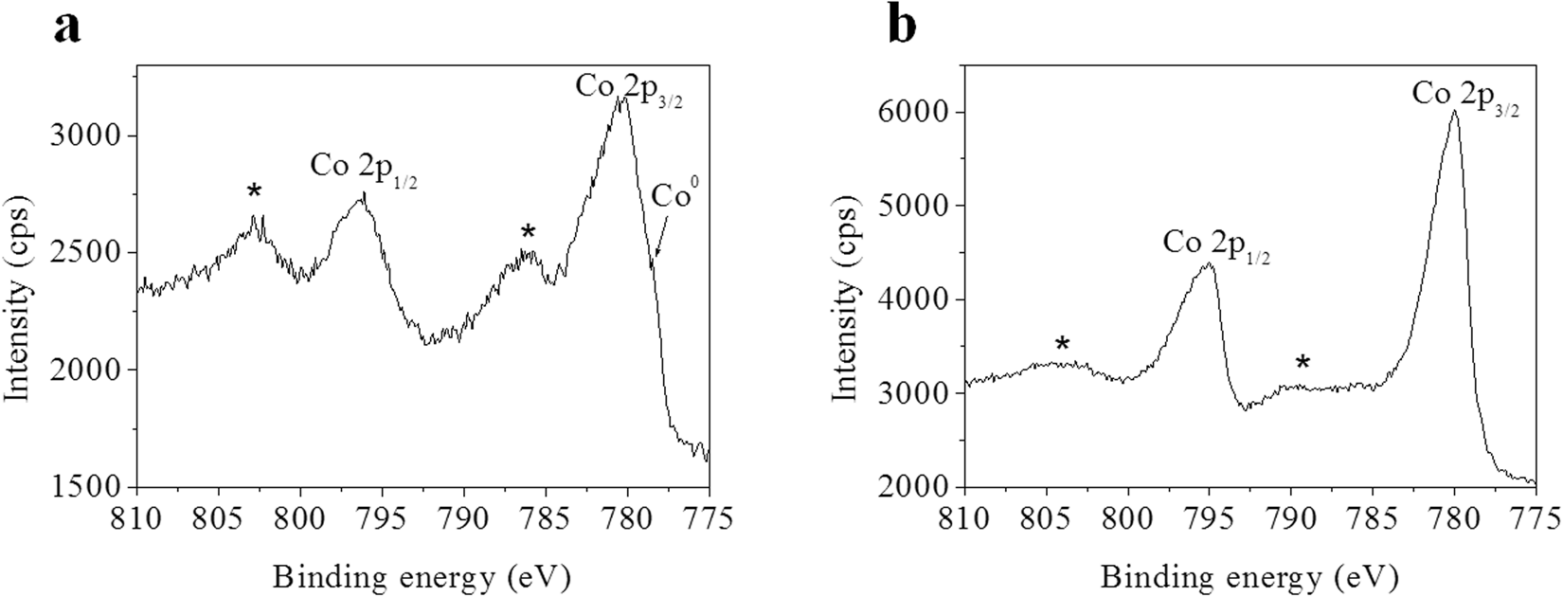
XPS Co 2p spectra of CoO_x_@CNTs/P900 electrode. (**a**) ‘fresh’ sample and (**b**) ‘spent’ sample (after 100 cycles) (*: satellite peaks; Co^0^: metallic cobalt).

The chemical nature of the cobalt oxide catalyst on CoO_x_@CNTs/P900 were revealed by different techniques such as XRD, HRTEM, Raman spectroscopy and XPS. The survey X-ray photoelectron spectrum of as synthesized CoO_x_@CNTs/P900 catalyst is shown in Supplementary Fig. S3a and reveals strong signals from cobalt, carbon and oxygen as well as a small nitrogen and sodium signal, as expected from the synthesis process of the carbon foam^19^. It is noteworthy that no signal of sulfur can be detected suggesting that thiophene was decomposed completely upon the annealing process. Figure 3a and b show the X-ray photoeletron spectra of CoO_x_@CNTs/P900, before and after OER testing. The presence of a doublet at 780.5/796.0 eV for Co 2p_3/2_ and Co 2p_1/2_, respectively is in line with the expected binding energies of Co 2p doublet in cobalt (II) oxide^28, 29^. Moreover, high intense satellite structures at 786.2 and 802.4 eV for Co 2p_3/2_ and Co 2p_1/2_, respectively suggests the presence of high-spin Co (II) ions at the surface. Interestingly a shoulder peak at around 778 eV can be attributed to metallic cobalt on the surface of the CNTs/P900 sample likely due to a reduction of cobalt to lower oxidation states at the high temperature treatment in inert atmosphere^29–31^. XRD and HRTEM analysis of the lattice fringes in the catalyst nanoparticles (Fig. 1d and e, respectively) suggests both CoO and Co_3_O_4_ phases present in the samples, in excellent agreement with electron diffraction measurements (Fig. 1f)^32, 33^. The Raman spectrum of CoO_x_@CNTs/P900 is vastly dominated by characteristic vibrations of crystalline Co_3_O_4_ with clear peaks at 482 cm^−1^ (E_g_), 519 and 621 cm^−1^ (F2_g_) and 690 cm^−1^ (A1_g_) (Supplementary Fig. S8b)^34–36^. Due to the strong Raman cross section of the Co_3_O_4_ peaks^37, 38^ and the much smaller Raman cross section of Co(II) oxides, the peaks at 455 and 675 cm^−1^ are almost fully hidden in the envelopes of the stronger Co_3_O_4_ signals^34^. In overall, based on these aforementioned evidences we can conclude that the CoO_x_@CNTs/P900 comprises a mixture of CoO, Co_3_O_4_ and very minute fraction of metallic cobalt on the surface of the sample after the annealing process.

X-ray photoelectron spectrum of the CoO_x_@CNTs/P900 after OER testing, as shown in Fig. 3b, reveals a significant change in oxidation state of cobalt manifested by a sharpening of the Co 2p_3/2_ and Co 2p_1/2_ peaks and the disappearance of the metallic cobalt signal as well as a dramatic loss in the intensity of the satellite peaks. This observation is similar with the phenomenon observed by Petitto and his coworkers in their XPS study of the transformation of Co (II) oxide to Co_3_O_4_ at high temperatures under oxygen atmosphere^28^. The oxidation of cobalt during the OER tests was further evidenced by comparing the O 1 s spectra of the”fresh” and”spent” catalysts (Supplementary Fig. S3c and d), revealing a clear increase in the intensity of the O 1 s component corresponding to Co = O bond at 529.7 eV for the sample subjected to 100 OER sweeps. It was further confirmed by smaller Co_total_/O_(529.7 eV)_ atomic ratio of the “spent” catalyst compared with that of the “fresh” catalyst (1.1 and 1.5, respectively). Thus, it is plausible that both the surface metallic cobalt and cobalt (II) oxide were further oxidized to form Co_3_O_4_ by newborn oxygen atoms formed during the OER process. Generally, after the electrochemical reaction, Co_3_O_4_, as also confirmed by the Raman spectroscopy (Supplementary Fig. S8b), was the dominant phase on the surface of the CoO_x_@CNTs/P900 electrode.

The electrocatalytic activities of CoO_x_@CNTs/P900 electrode and the “blank” reference (CNTs/P900) were tested in alkaline electrolyte (0.1 M KOH). As illustrated in Fig. 2a, the higher current density of the “blank” CNTs/P900 electrode clearly indicates that the electrode possesses a higher surface area compared to the “blank” P900. After growing CNTs, there is no sign of cobalt on the surface of CNTs/P900 sample (Supplementary Fig. S3a) indicating that the cobalt catalyst particles were completely covered by carbon. Thus, we can conclude that the contribution of the cobalt metal which was used as catalyst in CNTs growing process to the OER performance of CNTs/P900 and also CoO_x_@CNTs/P900 sample is very minor. On the other hand, the electrocatalytic activity of the CoO_x_@CNTs/P900 electrode was much better than that of the CoO_x_@P900 electrode giving rise to a lower overpotential (0.42 V compared with 0.53 V, at 10 mA/cm^2^, no iR corrected) as well as to a much higher current density. After growing CNTs, compared with the original bulk density of P900, the bulk density of the CNTs/P900 was almost doubled. Thus the absolute weight of cobalt oxide catalyst on CNTs/P900 sample, which has the same catalyst loading (16 wt. %) with CoO_x_@P900 sample, is two times higher than the amount of cobalt oxide on P900 sample. This could explain for the higher OER performance of CoO_x_@CNTs/P900 sample over CoO_x_@P900. Besides, the introduction of cobalt oxide nanoparticles on CNTs (Fig.1b and c) brought along a significantly higher active surface area and, consequently, a higher catalytic activity compared to the cobalt oxide film found on the CoO_x_@P900 electrode (Supplementary Fig. S5). Ahn *et al*. found that the OER activity and the bubble genesis behavior of a catalyst are dependent on the surface morphology^39^. Metal oxide catalysts with film-like morphology do not perform well upon electrolysis of water due to their low catalytic activity (low surface area) and the platelet morphology also hampers the bubbles ability to detach from a surface (increasing hydrophobicity). Consequently, a higher electrocatalytic activity was observed for the CoO_x_@CNTs/P900 electrode. As showed in the Table 2, eventhough the overpotential of CoO_x_@CNTs/P900 electrode (at 10 mA/cm^2^) is higher than some reported value in the literature, which used better catalysts *e.g*. NiFe or different material *e.g*. nickel foam, comparing with the results which based on cobalt oxide catalyst, our material showed a high OER performance (0.38 V vs. RHE) after iR compensation (Fig. 2b). Moreover, it is important to emphasis that the aim of our work was to introduce a robust carbon foam based electrocatalyst platform rather than finding the ultimate electrocatalyst for OER.

**Table 2:** OER activities of some electrocatalysts in 0.1 M KOH at a current density of 10 mA.cm^−2^.

Materials	Overpotential (mV)	References
CoO_x_@CNTs/P900	380	This work
Co_3_O_4_@NCNTs/CP	470	11
Co_3_O_4_/mMWCNT	390	42
Fe-Co_3_O_4_	486	43
Au/mCo_3_O_4_	440	44
20 wt% Ir/C	380	45
20 wt% Ru/C	390	45
Mn_3_O_4_/CoSe_2_	450	13
NiFe-LDH/CNT	308	46
LDH/oGSH	350	47
NiFe/NF	240	12

The stability test of CoO_x_@CNTs/P900 and also CoO_x_@P900 electrodes were carried out using a chronoamperometric mode at a constant potential (1.7 V vs RHE, 3 electrodes setup) in a 0.1 M KOH solution for around 5 h/cycle (total 2 cycles). As shown in Fig. 2c, while a large drop of the current density of CoO_x_@P900 electrode was observed, the current density of the CoO_x_@CNTs/P900 electrode only showed a minor downward trend after 5hrs in the first testing cycle. The deactivation of the catalyst could be due to several reasons such as: corrosion of the carbon substrate^40, 41^, degradation of the electrolytes (pH and conductivity change)^42^. It is evident that the corrosion of the carbon substrate can be the main reason for the deactivation of CoO_x_@P900 where much less of catalyst was found on the surface of the sample after the test (Supplementary Fig. S5b) and cobalt (at the wavelength of 228.616 nm) was found in the electrolyte after the stability test (Supplementary Fig. S10). Moreover, the electrocatalytic performance of the 2^nd^ round of CoO_x_@P900 electrode could not be recovered properly clearly implicating that irreversible corrosion of carbon happened. In the case of CoO_x_@CNTs/P900, there is no sign of cobalt was found in the electrolyte after stability test. The corrosion process can be remedied by the introduction of CNTs as well as cobalt oxide nanoparticles which are grown/decorated on the carbon foam surface. The fast transport of the generated electrons from the cobalt oxide nanoparticles to the CNTs/P900 surface should help to prevent charge accumulation on the surface and, subsequently, reduce unwanted corrosion reactions^42^. Also, cobalt oxide nanoparticles preferentially occupy most of the defective sites^11^ and oxygen containing groups which are more prone to the corrosion. The presence of CoO_x_ nanoparticles should counteract corrosion processes at the electrode surface^42–44^. Noteworthy, when the electrolyte in the electrochemical cell was replaced by a fresh electrolyte after the 1^st^ round, the chronoamperometric curve of the 2^nd^ round of CoO_x_@CNTs/P900 shows a very similar trend as the 1^st^ round (Fig. 2c) strongly indicating that the decrease in current density was most likely due to degradation of the electrolyte In summary, all of these evidences represented the excellent stability of the CoO_x_@CNTs/P900 electrode.

We speculate that the good electrocatalytic properties of CoO_x_@CNTs/P900 is rationalized by a combination of high electrical conductivity, a good charge transport in the vicinity of the active catalysts and a well-balanced morphology of the 3D electrode. The latter property is highly important since it will allow an efficient diffusion of gas bubbles throughout the whole electrode. It has been reported that attachment of gas bubbles on an electrode surface will result in lower electrocatalytic performance and the stability of electrodes, especially in case of a 2D electrode^11, 12^ by blocking the active catalyst sites and hindering ionic transport. As reported in the literature, the macroporous nickel foam with a pore size from 100 to 200 μm allows for a fast dissipation of large oxygen bubbles into the electrolyte^12^. Consequently, similar behavior can be anticipated from carbon foam which also possesses large pores, ranging from 50–100 μm (Supplementary Fig. S6a). Moreover, due to the flexibility of the carbon foam, upon wetting by water, the pores can be expanded and thus further facilitate the dissipation of oxygen bubbles. As shown in Fig. 2d, the CoO_x_@CNTs/P900 electrode was allowed to run for 100 LSV cycles (from 1–1.9 V vs RHE, scan rate of 5 mV/s in 0.1 M KOH solution). After 100 cycles, the anodic current manifested no decrease but even a bit higher catalytic activity than the current collected from the first cycle. The result strongly confirmed that the material is able to quickly dissipate the oxygen bubbles formed during OER of the CoO_x_@CNTs/P900 electrode.

## Conclusions

A novel 3-D hierarchical carbon foam and carbon nanotube structure was applied as anode in OER. Depending on the nature of the heat treatment process, the electrical conductivity of the carbon foam can be easily tuned. In reality, upon comparison of the electrical conductivity and the electrocatalytic activity, the P900 matrix is considered as a suitable material to be used directly as the catalyst support for cobalt oxide catalyst which in turn can be used as oxygen electrode in the water electrolysis process. Moreover, the surface area of the P900 sample can be enhanced up to 30 times by directly growing CNTs on the surface. Owing to the advantageous approach of directly growing CNTs as well as to directly decorate cobalt oxide catalyst on the carbon foam surface, the CoO_x_@CNTs/P900 electrode exhibited very high electrocatalytic activity also giving a low overpotential of only 0.38 V at 10 mA/cm^2^ and a good stability after 2 × 5 h under the testing condition. Additionally, due to its unique macroporous frame, the electrode was also able to quickly dispose the oxygen bubbles formed during the water electrolysis process. In summary and on the basis of the results obtained, we anticipate that our carbon foam material which can be synthesized through a simple, scalable and cost effective pyrolysis process is a potent candidate in industrial or large-scale production of electrodes for the water electrolysis.

## Electronic supplementary material

Supporting information
