# Supplementary material for: Robust hierarchical 3D carbon foam electrode for efficient water electrolysis
Source: Sci Rep. 2017 Jul 21;7:6112. doi: 10.1038/s41598-017-05215-1 (PMC5522430; doi:10.1038/s41598-017-05215-1)
Supplement: Supplementary file 1 — Supporting information [file 41598_2017_5215_MOESM1_ESM.doc]

**Supporting Information**

**Robust hierarchical 3D carbon foam electrode for efficient water electrolysis**

Tung Ngoc Pham^1,2^, Tiva Sharifi^3^, Robin Sandström^3^, William Siljebo^1^, Andrey Shchukarev^1^, Krisztian Kordas^4^, Thomas Wågberg^3^ and Jyri-Pekka Mikkola^1,5^

^1^Technical Chemistry, Department of Chemistry, Chemical-Biological Centre, Umeå University, SE-90187 Umeå , Sweden, ^2^ Department of Chemistry, The University of Danang, University of Science and Technology, 54 Nguyen Luong Bang, Lien Chieu, Da Nang, Viet Nam, ^3^Department of Physics, Umeå University, SE-90187 Umeå , Sweden,  ^4^Microelectronics and Materials Physics Laboratories, Department of Electrical Engineering, University of Oulu, P.O. Box 4500, FI-90014 University of Oulu, Finland, ^5^Industrial Chemistry & Reaction Engineering, Department of Chemical Engineering, Process Chemistry Centre, Åbo Akademi University, FI-20500, Åbo-Turku, Finland.

**Supplementary Figure S1.** (**a**) Precursor polymer foam (left) and carbon foam (right), (**b**) carbon foam electrode and (**c**) three electrode set-up.

**Supplementary Figure S2.** Current-Voltage sweep of A800, P900 and CNTs/P900 samples

**a**

**c**

**d**

Co=O

Co=O

**b**

**Supplementary Figure S3.** (**a**) XPS wide spectra of P900, CNTs/P900 and CoO_x_@CNTs/P900 samples, (**b**) N1s SPX spectra of P900 sample, O 1s XPS spectra of ‘fresh’ (**c**) and ‘spent’ (**d**) (after 100 LSV cycles) of CoO_x_@CNTs/P900.


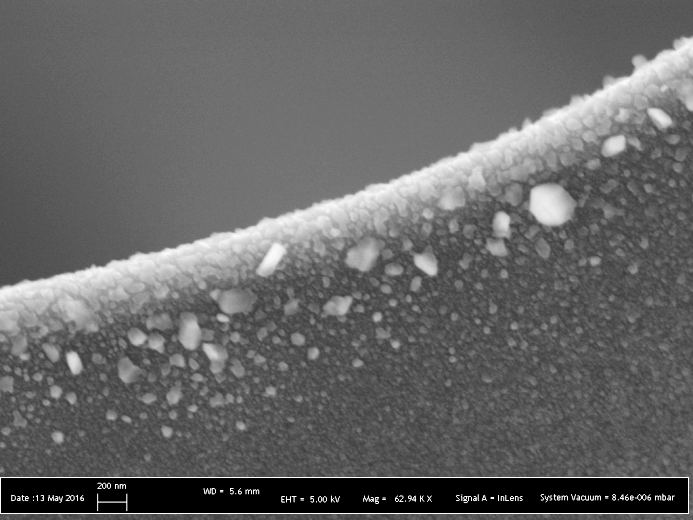

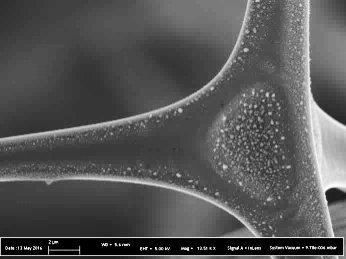


**Supplementary Figure S4.** SEM image of CoO_x_@A800. Scale bar, 200 nm, inset 2 μm.


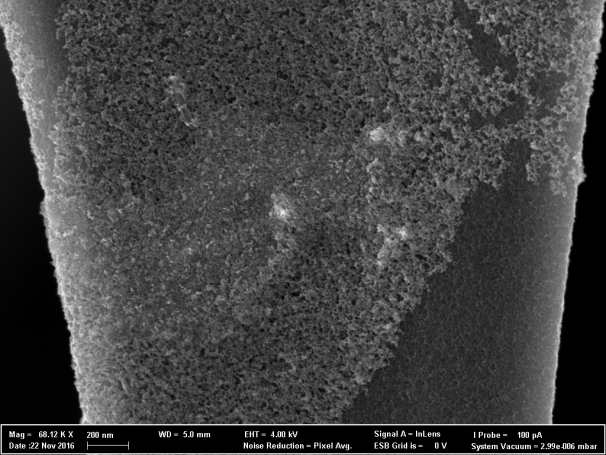

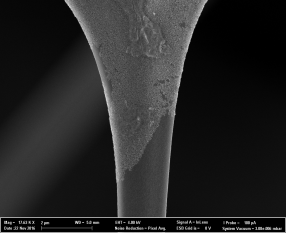

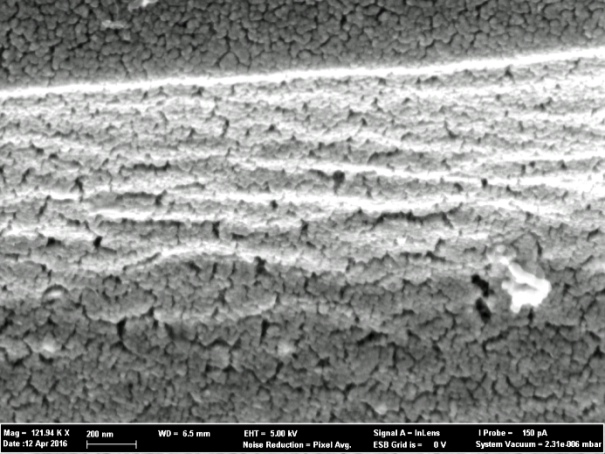

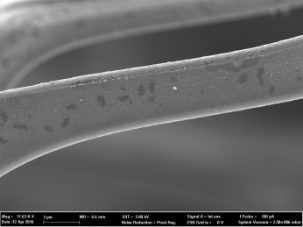


**a**

**b**

**Supplementary Figure S5.** SEM image of CoO_x_@P900, before (**a**) and after (**b**) stability test. Sale bar, 200 nm, inset 2 μm.

**Supplementary Figure S6.** (**a**) SEM image and (**b**) TEM images of CoO_x_@CNTs/P900. Scale bars, 10 µm (**a**), 500 nm (**b**).


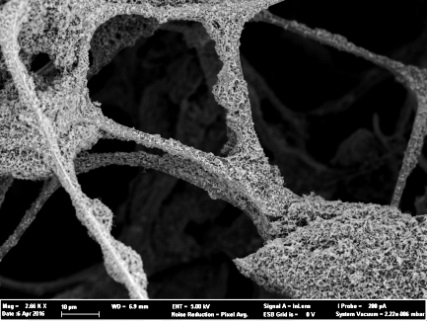


**a**


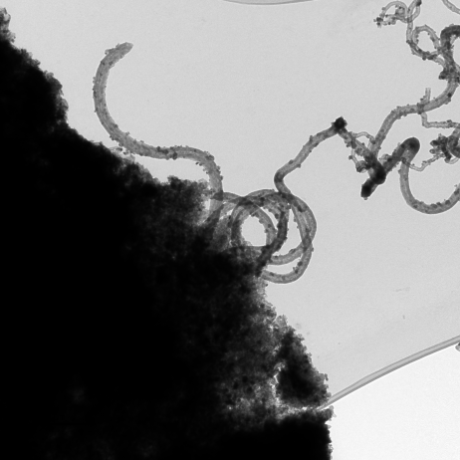


**b**

**Supplementary Figure S7.** Particle size distribution of cobalt coxide nano particles on the surface of CoO_x_@CNTs/P900.

**a**


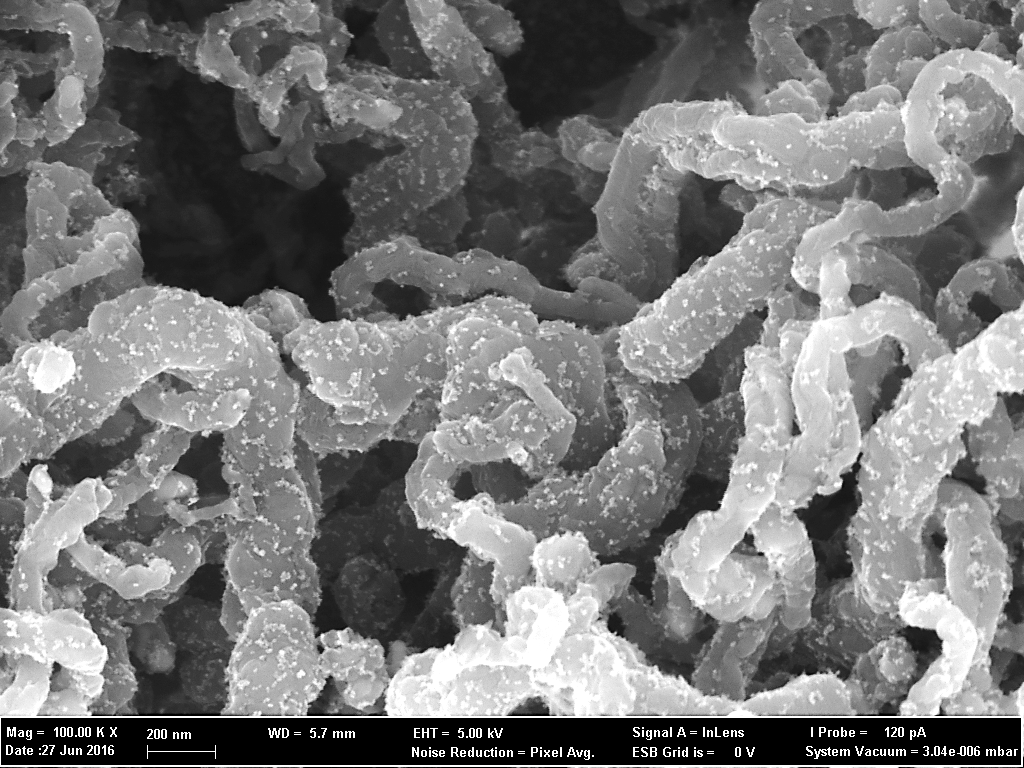


**b**

**Supplementary Figure S8.** (**a**) SEM image of CoO_x_@CNTs/P900 (after stability test in 10 h, Scale bar: 100 nm) and (**b**) Raman spectra of “fresh” (bottom) and “spent” (top, after 100 LSV cycles) of CoO_x_@CNTs/P900.


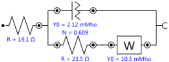


R_ct_

R_s_

R_s_

R_ct_

**Supplementary Figure S9.** The impedance spectroscopy spectra of (**a**) CoO_x_@CNTs/P900 (**b**) CNTs/P900 and P900 electrodes recorded at 0 V vs. Ag/AgCl in 0.1 M KOH. Inset: zoom in view of the Nyquist plot.

**Supplementary Figure S10.** Determination of cobalt in the electrolyte after the stability test of CoO_x_@P900 and CoO_x_@CNTs/P900 by ICP-OES.
